# Investigating the Dewatering Efficiency of Sewage Sludge with Optimized Ratios of Electrolytic Manganese Residue Components

**DOI:** 10.3390/ma17143605

**Published:** 2024-07-22

**Authors:** Xuquan Huang, Jun Wang, Fei Xue, Xiaorong Zhao, Ziyao Shi, Qingyang Liang, Haojie Wang, Ziyu Zhao

**Affiliations:** 1 Engineering Research Center of Eco-Environment in Three Gorges Reservoir Region, Ministry of Education, China Three Gorges University, Yichang 443002, China; hxq@ctgu.edu.cn (X.H.); rongrong315@ctgu.edu.cn (X.Z.); wanghaojie@ctgu.edu.cn (H.W.); 2 College of Hydraulic and Environmental Engineering, China Three Gorges University, Yichang 443002, China; 3 Key Laboratory of Solid Waste Disposal and Resource Utilization, China Three Gorges University, Yichang 443002, China; 4 School of Environment and Architecture, University of Shanghai for Science and Technology, Shanghai 200093, China

**Keywords:** electrolytic manganese residue, sewage sludge, dewatering

## Abstract

As an industrial waste residue, Electrolytic Manganese Residue (EMR) can greatly promote sludge dewatering and further particle-size optimization can significantly strengthen sludge dewaterability. In this study, the effects of ammonium sulfate, calcium sulphate dihydrate, and manganese carbonate in EMR on sludge dewatering performance were investigated using the response surface optimization method. It was found that the optimized ratio of three components in EMR was 1.0:1.6:2.2 based on capillary suction time (CST), specific resistance of filtration (SRF), and zeta potential of dewatered sludge. The composition ratio of particle-size optimized EMR was modified based on the above optimization, resulting in a significant increase in sludge dewatering performance (CST and SRF reduced by 8.7% and 11.2%, respectively). Compared with those in original sludge, the content of bound extracellular polymeric substances in the conditioned sludge with optimized ratio was drastically reduced while that of soluble extracellular polymeric substances was slightly increased, which was in accordance with the decline of fluorescence intensity. These findings indicated the disintegration of extracellular polymeric substances, the enhancement of hydrophobicity, and dewatering properties of the sludge. In summary, optimized EMR can effectively intensify the dewaterability of sludge, providing a competitive solution for dewatering and further disposal of sludge.

## 1. Introduction

The generation of overabundant activated sludge is unavoidable [1] with the widely used activated sludge process in wastewater treatment. With the rapid development of industrialization and urbanization, vast waste activated sludge, as a by-product containing complex pollutants, have originated from wastewater treatment plants worldwide [2]. These large quantities of sludge commonly contain over 90% water. Deep dewatering (to reduce the water content less than 75%) can significantly decrease the mass and volume of sludge for subsequent treatment and disposal [3]. Thus, different kinds of methods such as sludge dewatering [4], thermal drying [5], and granulation for fuel preparation [6] have been employed to realize sludge reduction. Among the currently available sludge reduction methods, sludge dewatering is an important option for saving energy and improving effectiveness.

The findings of existing research show that the rheological properties, particle size, microstructure and porosity, surface charge and repulsive energy, and extracellular polymeric substances (EPS) are important factors influencing sludge dewatering [7]. However, the composition of sludge is complex, and the surface charge of sludge particles is negative due to the wrapping by EPS [8]. EPS hinders the conversion of non-free water molecules to free water molecules, where large amounts of non-free water molecules are wrapped by organic matter around sludge particles and cannot convert into free water molecules [9]. Mechanical dehydration such as pressure filtration, vacuum filtration, and centrifugation, cannot effectively destroy EPS in sludge and is prone to introduce blocking in the filter cake due to the compressibility of the sludge particles, making the entire dehydration process less efficient [10]. Nowadays, physical conditioners and chemical conditioners are widely used before mechanical dehydration; in most cases, the addition of a physical conditioner leads to flocculation or coagulation induced by a chemical conditioner. Physical conditioners, often referred to as filter aids or skeleton builders due to their roles in sludge dehydration, are usually used to compress sludge and enhance the mechanical strength and permeability of the sludge filter cake [10]. Nowadays, different kinds of solid wastes have been employed as physical conditioners to intensity sludge dewaterability. Gahlot [5] has developed modified kaolin as a physical conditioner for sludge treatment and the treatment can reduce the water content of sludge to about 58%; however, the modification of kaolin needed heat treatment and acidification, increasing the difficulty and cost of practical application. Fly ash [11] and rice husk [12] have been employed as physical conditioners to intensity the dehydration of sludge; both required additional flocculants to help build the skeletons, which would make the sludge composition complex and increase the cost in practical applications as well. Therefore, it is urgent to develop a physical conditioner with prominent dewaterability and convenient usage.

Electrolytic manganese metal, as an important basic substance, is widely used in metallurgy, the chemical industry, the food industry, aerospace, and so on. Electrolytic Manganese Residue (EMR) is the byproduct of Electrolytic manganese metal production, in which manganese rhodochrosite (MnCO_3_) is acid-soluble processed by concentrated sulfuric acid and subsequently neutralized by ammonia [13], and it contains soluble sulfate [14], high concentration of ammonia nitrogen, and soluble heavy metals such as manganese, cadmium, and chromium [15]; long-term stockpiling of EMR is tough on the ecological environment [16]. Nowadays, about 20 million tons per year of EMR has been produced in China, and the total accumulated amount of EMR has come to about 130 million tons [17]. However, the comprehensive utilization ratio of EMR in China is less than 7% [18], and relevant studies on reutilization of EMR mainly focus on the brickmaking [19], cement materials [20]), epoxy resin [21], adsorbents [22], fertilizers [23], and recovery of valuable metals [24], etc. There are hardly any other studies about deep dehydration of sewage sludge with EMR as the conditioner.

Our group’s latest work on the deep dehydration of sludge has been successful with EMR as a physical conditioner and found that grain-size modification of EMR significantly contributed to the dewater ability of sludge [25]. Compared with that of initial sludge, the water content of sludge conditioned with grain-size modified EMR was decreased by 22.4%. Simultaneously, we also found that different proportions of optimized EMR (OEMR) components at the same particle size also had significant effects on the dehydration rate of sludge. Based on the preliminary work [25], we screened the EMR for granularity and named the screened EMR as OEMR; the contribution of the main components in the EMR to the dewaterability was further explored in this paper. The results show that all three lattice components, that is (NH_4_)_2_SO_4_, CaSO_4_·2H_2_O, and MnCO_3_, have a significant impact on the reinforcement of sludge dehydration. Another interesting finding is that some components in EMR can improve the specific resistance of filtration (SRF) of the filter cake and simultaneously neutralize the surface charge of the sludge particles as chemical conditioners. In this paper, we employed response surface methodology to optimize the matching rate of effective constituents in EMR to effectively destroy the EPS and significantly promote sludge dehydration effectiveness.

## 2. Materials and Methods

### 2.1. Experimental Materials

(NH4)_2_SO_4_, CaSO_4_·2H_2_O, and MnCO_3_, denoted as OEMR1, OEMR2, and OEMR3, respectively, were purchased from Hubei Tian Li Co., Ltd. (Yichang, China). The original sludge was obtained from Shahe Urban Sewage Disposal Works in Yichang City, Hubei Province, China, which treats wastewater (60,000 m^3^·d^−1^) using an Anaerobic-Anoxic-Oxic process. For the original sludge, the water content (wt%) and pH was 95 ± 3 and 8.27, respectively, and the capillary suction time (CST) and SRF was 125.2 s and 2.889 10^8^ s^2^·g^−1^, respectively. The chemical constitution of the dried original sludge and original EMR is given in Table 1 and Table 2, respectively. In this paper, the initial sludge without any conditioning is noted as A0.

### 2.2. Characterization of Sludge Dewatering Performance

We employed CST, SRF, wt%, and zeta potential as the pointers to appraise the sludge dehydration performances.

(1) CST test

With the aid of funnel and qualitative filter paper, the CST was determined with a DFC-10A capillary suction timer. More specific details about the characterization of sludge dewatering performance were referred to the experimental part of our group’s preliminary research work [25].

(2) SRF test

The SRF indicates the resistance of unit mass of sludge for a unit filtration area at a given pressure during the pressure filtration, and we detected it with the vacuum filtration method [26].

(3) wt% test

In this work, we use a small press filter during the sludge press filtration process to collect water in the bottom assemblies and an electric thermostatic drying oven in desiccation to conjointly determine the wt%, which is the ratio of total water mass in sludge vs. total mass of initial sludge.

(4) Zeta Potential Test

Take 30 mL of homogenized sludge sample and place it in a 50 mL centrifuge tube. After 15 min of water bath ultrasound, place it in a high-speed centrifuge for centrifugation treatment. Centrifuge at 8000× *g* for 15 min before taking out the supernatant zeta potential measurement. Each sludge sample needs to be tested three times for zeta potential testing, and finally using the average value as the result of this analysis.

### 2.3. Extraction and Determination of EPS

The employed EPS extraction method refers to the formaldehyde-sodium hydroxide extraction method [27], through which soluble EPS (S-EPS) and bound EPS (B-EPS) can be obtained, respectively. The EPS samples used in this paper were filtered through a microporous membrane (0.45-μm) to remove the residue, and then stored into a refrigerator at 4 °C. We analyzed the proteins (PN) by the modified Lowry method with bovine serum albumin as the standard [28]. The polysaccharides (PS) content was tested with the anthrone method with glucose as the standard [29].

### 2.4. Analytical Methods

The isothermal adsorption-desorption curve, distribution of pore diameter, and specific surface area test were all carried out on a SORP-max (MicrotracBEL Japan, Inc., Osaka, Japan). The zeta potential analyzer used in this paper was Beckman Coulter Delsa^TM^ Nano (Brea, CA, USA).

We measured the fluorescence excitation-emission-matrix Spectra (EEMs) on a F4600 Hitachi fluorescence spectrophotometer (Varian Eclipse, Bejing, China) in scanning mode. We gathered the EEM spectra with scanning emission Em spectra from 250 to 550 nm at an increment of 5-nm by regulating and controlling the excitation Ex wavelength from 220 to 350 nm at an increment of 5-nm. The spectra were recorded at a scanning rate of 4800 nm min^−1^, with 5-nm slit bandwidths of excitation and emission.

## 3. Results and Discussion

### 3.1. Result and Analysis of Box-Behnken Test

To optimize the material dosage in the process of the dewaterability of the sludge, 17 experimental sets (Tables S1–S4 in the Supplementary Materials) were employed for response surface modeling with CST, SRF, and zeta potential as the response value. Data fitting and regression analysis were carried out with Design Expert 8.06 for 17 experiments of design matrix, respectively. The fitting polynomial Equation of CST (1), SRF (2) and zeta potential (3) obtained as follows:

Y_1_ = 82.87 − 8.69A − 21.99B + 3.39C + 2.49AB − 0.77AC + 2.22BC + 3.31A^2^ + 2.31B^2^ − 3.64C^2^, (1)

Y_2_ = 0.74 + 0.045A + 0.026B − 0.22C − 0.050AB + 0.033AC + 0.026BC + 0.10A^2^ + 0.074B^2^ − 0.081C^2^, (2)

Y_3_ = −10.08 − 0.81A − 0.76B + 1.95C + 0.39AB − 0.59AC + 0.50BC + 0.81A^2^ − 1.37B^2^ + 1.55C^2^, (3)

where Y_1_ is CST value (s), Y_2_ is SRF value (×10^8^ s^2^·g^−1^), Y_3_ is zeta potential (mV), and A, B, and C is the dosage of OEMR1, OEMR2 and OEMR3 (g/100g sludge), respectively.

It can be seen from the above three equations that the three factors, including (NH_4_)_2_SO_4_, CaSO_4_·2H_2_O, and MnCO_3_ in the EMR, have an interactive effect on the CST, SRF, and zeta potential of the sludge. In order to verify the accuracy and reliability of the model, we carried out ANOVA and correlation analysis, and the detailed results are presented in Table 3, Table 4, Table 5 and Table 6 Compared with those of coefficients for OEMR1 and OEMR2, the values of coefficients for OEMR3 are much bigger according to the above Equations (1) and (3), indicating the significance of this parameter in both Y_1_ model (Equation (1)) and Y_3_ model (Equation (3)).

As shown in Table 3, ANOVA for quadratic model of the CST was performed to evaluate the importance. The “*p*-value” (Prob > F) (far less than 0.0500) and the “Model F-Value” of 75.08 reflected this model was statistically significant [30]. The model’s veracity could also be validated by the F-value of item “Lack of Fit”, and the F-value of item “Lack of Fit” (3.68) in Table 4 implies the item “Lack of Fit” was not significant relative to the pure error. Low values of coefficient of variance (C.V. %) for CST confirmed high repeatability of the experimental results [31]. In addition, the “Adeq Precision” measures the ratio of signal vs. noise and the “Adeq Precision” of 33.368 in this model implied that the proposed models have high fitting degree and sufficient signal-to-noise. The high correlation coefficient (R-Squared) value of 0.9897 for CST indicated that the regression models are significant. A negative “Pred R-Squared” also implied that the overall mean is a better predictor of response parameter than the current model [32].

Generally, the greater the mean square value, the more sensitive the response value varied with influence factor is [33]. However, in this study, the smaller the CST value is, the better the sludge dewatering performance is [34]. Therefore, the smaller the CST value is, the less the mean square is, and the better the dewatering performance of sludge is.

According to the results in Table 4, it can be concluded that the significance order of influence among A, B, and C on the CST of sludge is C > A > B. Therefore, the variation of C value would significantly influence the dewatering performance, but that of B would have relatively light influence on the dewatering performance.

**Figure 1:**
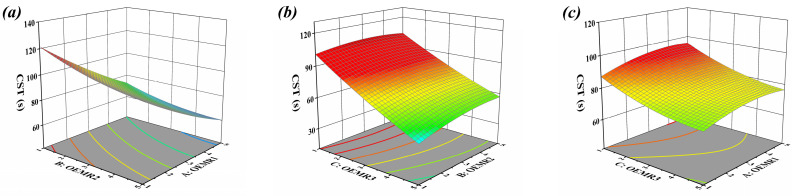
Response surfaces of different conditioners to CST of conditioned sludge: (**a**) interaction of OEMR1 and OEMR2, (**b**) interaction of OEMR2 and OEMR3, (**c**) interaction of OEMR1 and OEMR3.

The 3D response surface plots described by the regression model show the effect of independent variables and the interaction of independent variables on the CST. The dosage of OEMR3 in Figure 1a, OEMR1 in Figure 1b, and OEMR2 in Figure 1c was set as 3%, respectively. The graphs in Figure 1a,b are inclined slopes, indicating that the interaction between the two factors is not significant [35]. Conclusions are drawn based on the results of the response surface calculation that the three influencing factors have a positive impact on the CST value of sludge in the order of C > A > B.

The 3D response surface of the regression model appears obliquely planar, which indicates that the relationship between one argument and the corresponding value tends to be linear while the other argument remains constant. It can be seen from Figure 1a that when the dosage of factor A was 3%, the smaller the CST value with the gradually increasing dosage of factor B, the better the dehydration performance of the sludge. However, when the content of factor B is 3%, with the gradual increase of the content of factor A, the change of CST value has the same pattern as the former, but the decrease of CST value at this time was obviously weakened. These results show that factor A has a more significant effect on the CST value under the same conditions. From Figure 1b,c, as can be obtained in the same way according to the inclination angle of the graph, the influence of factor C on the CST value of sludge is more significant than that of factor B and A. The above conclusions are consistent with the results of the response surface model calculating in Table 4.

ANOVA for quadratic model of the SRF value was performed to evaluate the importance (Table 4). The *p*-value (Prob > F) was far less than 0.0100, indicating that the model has reached a very significant level. The *p*-value of the item “Lack of Fit” was much bigger than 0.0500, indicating the small difference of the item “Lack of Fit” of the model, which can reflect the actual situation; in other words, the regression model is appropriate. The value of R-Squared and Adj R-Squared is 0.9868 and 0.9699, respectively, indicating that only 3.01% of the SRF value cannot be predicted by this model. The coefficient of variation (C. V. %, 3.93%) in the model indicates that the model fits well with the actual situation of the test and can effectively reflects the real experimental value, so the reliability of the model is high. The smaller the SRF value of the sludge, the better the dewatering performance of the sludge. Therefore, the smaller the Mean Square value, the more sensitive the influence factor is.

**Figure 2:**
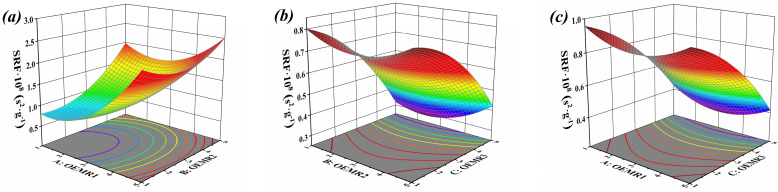
Response surfaces of different conditioners to SRF of conditioned sludge: (**a**) interaction of OEMR1 and OEMR2, (**b**) interaction of OEMR2 and OEMR3, (**c**) interaction of OEMR1 and OEMR3.

The 3D response surface plots described by the regression model reveals the effect of independent variables and the interaction of independent variables on the SRF. The dosages of OEMR3 in Figure 2a, OEMR1 in Figure 2b, and OEMR2 in Figure 2c were set as 3%, respectively.

The response surface corresponding with the quadratic regression Equation (2) is a concave surface with an opening upward, indicating that there is a minimum value of the response value (SRF value) within the given investigation range. When the 2D contour plot was projected onto the gray bottom of the 3D plot, an obvious ellipse appeared (Figure 2a). If the contour line was elliptical, the interaction between the two factors would be strong [33], so it can be concluded that the interaction between factor A and factor B is the most obvious. The greater the curvature of the 3D curve appears, the more obvious interaction between the two factors will be [33]. The curvature degree of the two 3D surfaces was similar but not obvious (Figure 2b,c), and the contour map also presented similar distribution patterns, so it can be inferred that the interaction between BC factors and AC factors on the sludge SRF values is not significant.

ANOVA for quadratic model of the zeta potential value was performed to evaluate the importance (Table 5). The *p*-value (Prob > F) of the model is 0.0051 (much less than 0.0500), indicating the significance of this model. The *p*-value of the item “Lack of Fit” is 0.0821 > 0.0500, indicating that the difference of the item “Lack of Fit” in this model is not significant, which can reflect reality; in other words, the regression model is appropriate. From the mean square value, it can be seen that the influence of three factors on the zeta potential value of the sludge is in the order: B < A < C.

**Figure 3:**
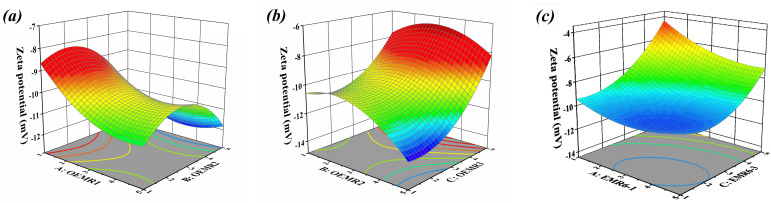
Response surfaces of different conditioners to zeta potential of conditioned sludge: (**a**) interaction of OEMR1 and OEMR2, (**b**) interaction of OEMR2 and OEMR3, (**c**) interaction of OEMR1 and OEMR3.

As mentioned above, the 3D response surface plots described by the regression model present the effect of independent variables and the interaction of each independent variable on the zeta potential. The dosages of OEMR3 in Figure 3a, OEMR1 in Figure 3b, and OEMR2 in Figure 3c were also set at 3%, respectively.

An obvious ellipse emerges in the two-dimensional contour map (Figure 3c), which indicates that factors A and C have a significant interaction on the increase of zeta potential value of the sludge. The 3D figures in Figure 3a,b show an upwardly arched saddle shape, indicating a certain interaction between factor A and B in Figure 3a or between factor B and C in Figure 3b. The change of graph surface in Figure 3b is much greater than that in Figure 3a, implying that the interaction of factor B and C is stronger than that of factor A and B. From the *p*-values with the interaction of AB, AC, and BC (Table 6), it can be concluded that the *p*-value with factors interaction was in the turn of AC < BC < AB, which is consistent with the law of surface bending in Figure 3 above.

The OEMR of different component proportions acquired from the optimization with CST, SRF, and zeta potential as the dependent variables, respectively, were named as PA-1, PA-2, and PA-3, respectively. While PA-4 is the OEMR of optimized component proportion obtained with a set of constraints, that includes CST and SRF as the minimum values and zeta potential as the target value of 0 mV. The specific optimization results are shown in Table 6.

### 3.2. XRD Analysis with Internal Standard Method by Blending EMR Components

**Figure 4:**
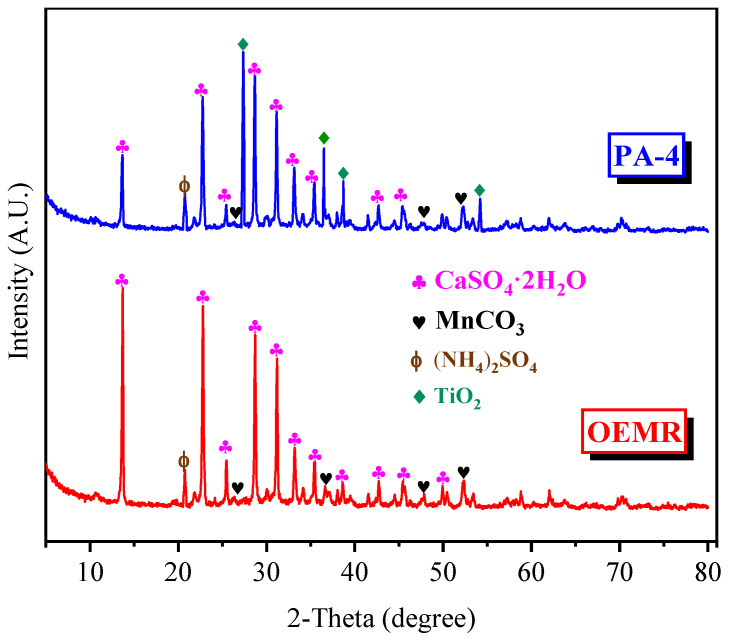
XRD patterns of OEMR and PA-4.

Because of the high purity and stable physicochemical properties, the rutile (TiO_2_) was employed as the internal standard to carry out the physical phase analysis and quantitative calculation of the samples. In this work, the rutile powder and the powder sample were fully mixed at a mass ratio of 3:7 and ground to a complete mixture, and then the XRD patterns of the composite sample with the internal standard was obtained by XRD scanning. The corresponding phases were searched in software Jade (6.0), and then the content of the internal standard in the crystal was calculated by software Maud (2.12). The results of XRD analysis of OEMR and PA-4 are demonstrated in Figure 4 and those of the quantitative analysis are shown in Table 7.

It can be seen that the main substances and mineral phases in the OEMR are gypsum (CaSO_4_·2H_2_O), manganese carbonate (MnCO_3_), and Ammonium sulfate ((NH_4_)_2_SO_4_) (Figure 4). Three diffraction peaks similar to OEMR appear in PA-4 and the diffraction peaks of internal standard rutile (TiO_2_) appear clearly.

The crystal structure models of the three main phases in OEMR were imported into Maud. The diffraction patterns were refined in Maud for various parameters (such as scale factor, phase parameters, crystal parameters, etc.), and the least squares method was used to continuously adjust the pattern correction parameters to make the computer theoretical patterns approximate to the real measurement patterns. Finally, the quantitative results of the phases were obtained by calculation. From Table 7, we can see the distribution of OEMR1, OEMR2, and OEMR3 in OEMR and PA-4, where OEMR1:OEMR2:OEMR3 = 1.0:1.5:2.4 in PA-4, which is very close to the target value (1.0:1.6:2.2).

### 3.3. Dehydration Test of Response Surface Optimization Group with a Filter Press

**Figure 5:**
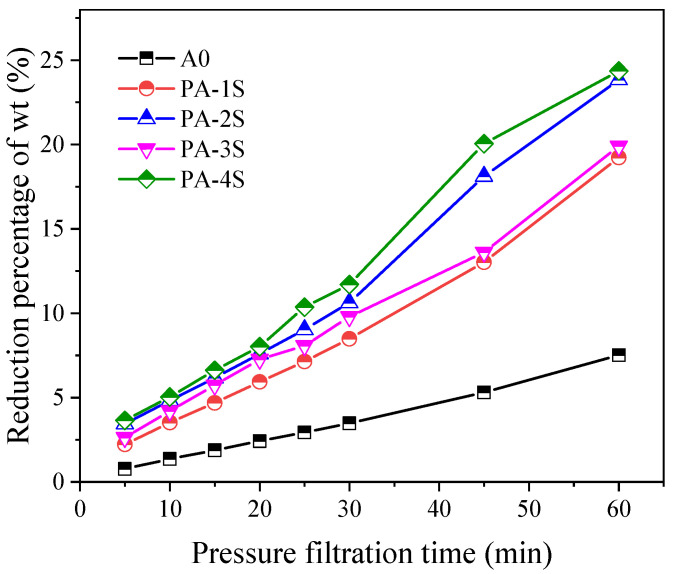
Variation curve of dehydration ratio of optimal performance group.

The sludges conditioned with PA-1, PA-2, PA-3, and PA-4 are named as PA-1S, PA-2S, PA-3S, and PA-4S, respectively. The dehydration rate of A0 was less than 10%, while that of PA-1S and PA-3S could reach 20% under the same conditions, and that of PA-2S and PA-4S touched 25% (Figure 5). Among them, PA-4S showed the best performance in the dewatering process. The dehydration rate of PA-1S, PA-2S, PA-3S, and PA-4S held the similar trend in the first 30 min, but that of PA-3S and PA-4S increased significantly after 30 min and continued the dehydration advantage until 60 min. Throughout the dehydration process, PA-4S performed slightly better than PA-3S in the dehydration rate. It can be concluded that PA-4S has the best dewatering effect by filter press according to the moisture content reduction curve.

### 3.4. Distributions and Compositions of EPS

#### 3.4.1. Concentration Distribution of Different Components in EPS

**Figure 6:**
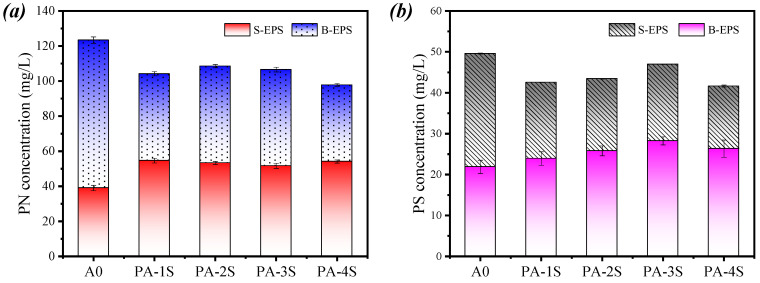
Effect of different conditioning agents on (**a**) PN and (**b**) PS in S-EPS and B-EPS of sludge.

The proteins (PN) and polysaccharides (PS) are the principal component of the EPS matrix in the activated sludge, containing a large quantity of functional groups. Figure 6 shows the changes of PN and PS contents in S–EPS (Soluble-Extracellular Polymeric Substances) and B–EPS (Bound-Extracellular Polymeric Substances), respectively. The PN of S-EPS increased in all four groups of filtrates obtained from the conditioned sludge, and that of B-EPS decreased (Figure 6a), which indicates that the use of PA-1, PA-2, PA-3, and PA-4 can effectively cleave B-EPS to S-EPS, thus, releasing the bound water wrapped around B-EPS and therefore increasing the content of S-EPS. Compared with that in A0, the PN of S-EPS in PA-1S, PA-2S, PA-3S, and PA-4S was increased by 36.50%, 33.20%, 29.10%, and 35.33%, respectively, indicating that the conversion rate of S-EPS in PA-1S reached the peak. The PN in B-EPS decreased by 16.25%, 12.75%, 14.31%, and 21.40%, respectively. Wilen [36] found that high abundance of protein may deteriorate the dewatering performance of the sludge because of its hydrophilicity and negative charges. Contrary to those of PN, the concentration changes of PS in S-EPS and B-EPS showed a different trend. The PS content of S-EPS in A0 was the highest, and that of S-EPS in PA-1S, PA-2S, PA-3S, and PA-4S increased by 9.32%, 17.91%, 29.05%, and 20.33%, respectively, while the PS in B-EPS decreased by 14.21%, 12.40%, 5.24%, and 16.01%, respectively. In general, the change of PN is more significant than that of PS. These findings indicate that the use of PA series conditioners can effectively decompose PN in B-EPS, but weakly decompose PS in B-EPS.

#### 3.4.2. 3D-EEM Fluorescence Spectra of EPS at Different Conditioner

**Figure 7:**
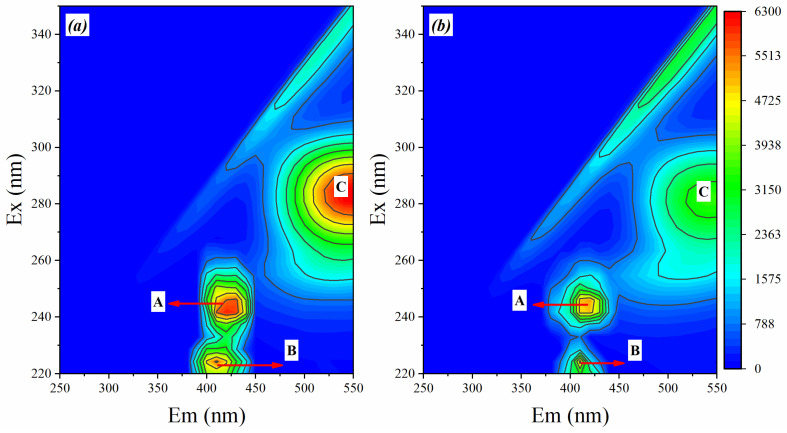
Excitation emission matrix (EEMs) profile of B-EPS fractions in (**a**) A0 and (**b**) PA-4S.

The organic compounds in the EPS of sludge samples were characterized by 3D-EEM fluorescence spectra, as shown in Figure 6. The X-axis represents the emission spectrum from 250 to 550 nm, while the Y-axis represents the excitation wavelength from 220 to 350 nm. Peak A was located in the region of the excitation/emission wavelengths (Ex/Em) between 255–265/315–330 nm, and Peak B was situated between 220–230/325–335 nm in Figure 7a. Peak A and Peak B were assigned to tryptophan-like substances and tyrosine-like substances, respectively [37], which illustrated that the tryptophan-like (Peak A) and tyrosine-like substances (Peak B) were two major substances in B-EPS in both A0 and PA-4S. Peak C located in the region of Ex/Em between 275–285/500–550 nm was presumed to be aromatic protein [8]. The fluorescence intensity of peak C in Figure 7b nearly doubles that of peak C in Figure 7a, indicating that the concentration of aromatic protein in Figure 7b is much smaller than that in Figure 7a. These findings strongly support the results presented in Figure 6. In addition, Peak A, Peak B and Peak C also appeared in the same region in Figure 7b, but the fluorescence intensity was much weaker than that in Figure 7a, which should be attributed to the reduction of protein content. These results reconfirm the conclusion in Figure 6.

## 4. Conclusions

In our preliminary work, the particle size modification of EMR has been proved to improve the dewatering properties of sludge. In this study, the effect of EMR component matching on deep dehydration of sludge was evaluated to screen the OEMR with optimum ratio (PA-4) and reveal the mechanism of deep dehydration of PA-4S with the EPS distribution and composition.

A response surface optimization method was employed to establish the regression relationship between three main components in EMR and sludge dewatering performance through regression analysis to obtain the optimal conditioner formulation, i.e., OEMR1:OEMR2:OEMR3 = 1.0:1.6:2.2.

Compared with those of A0, the contents of PN and PS in S-EPS and B-EPS of PA-4S were significantly reduced and the fluorescence intensity of PA-4S in EEMs was also much lower than that in A0. These results demonstrated that the significant improvement of dewatering performance of PA-4S was attributed to the destruction of EPS.

Overall, PA-4 has a positive contribution to the deep dewatering of sludge, as the result of a combination of particle size optimization and composition match. The results of this study may not only provide a solution to competitive sludge dewatering problems, but also make full use of hazardous solid wastes such as EMR.

## Figures and Tables

**Table 1:** Chemical constitution of dried original sludge (%).

Name	Al_2_O_3_	SiO_2_	Fe_2_O_3_	P_2_O_5_	CaO	SO_3_	K_2_O	TiO_2_	Cl	MnO	ZnO	V_2_O_5_
value	46.73	24.36	10.33	7.57	5.57	2.59	0.95	0.92	0.26	0.19	0.16	0.11

**Table 2:** Chemical constitution of the original EMR [25] (%).

Name	Al_2_O_3_	SiO_2_	Fe_2_O_3_	P_2_O_5_	CaO	SO_3_	K_2_O	TiO_2_	MgO	MnO	ZnO	SrO
value	8.812	29.04	4.022	1.087	5.929	19.494	2.171	0.398	1.82	2.976	0.011	0.022

**Table 3:** ANOVA results of Box-Behnken experiments with CST as the response parameter.

Source	Sum of Squares	df	Mean Square	F Value	*p*-Value (Prob > F)
Model	4729.48	9	525.50	75.08	<0.0001
A	603.61	1	603.61	86.24	<0.0001
B	3868.04	1	3868.04	552.64	<0.0001
C	92.07	1	92.07	13.15	0.0084
AB	24.70	1	24.70	3.53	0.1024
AC	2.36	1	2.36	0.34	0.5800
BC	19.67	1	19.67	2.81	0.1376
A^2^	46.14	1	46.14	6.59	0.0371
B^2^	22.48	1	22.48	3.21	0.1162
/C^2^	55.70	1	55.70	7.96	0.0257
Residual	48.99	7	7.00		
Lack of Fit	35.95	3	11.98	3.68	0.1204
Pure Error	13.04	4	3.26		
Cor Total	4778.47	16			
Std. Dev.	2.65	R-Squared	0.9897		
Mean	83.81	Adj R-Squared	0.9766		
C. V. %	3.16	Pred R-Squared	0.8754		
PRESS	595.63	Adeq Precisior	33.368		

**Table 4:** ANOVA results of Box-Behnken experiments with SRF as the response parameter.

Source	Sum of Squares	df	Mean Square	F Value	*p*-Value (Prob > F)
Model	0.50	9	0.055	58.26	<0.0001
A	0.016	1	0.016	16.71	0.0046
B	5.500 × 10^−^³	1	5.500 × 10^−^³	5.78	0.0472
C	0.37	1	0.37	389.17	<0.0001
AB	9.947 × 10^−^³	1	9.947 × 10^−^³	10.45	0.0144
AC	4.391 × 10^−^³	1	4.391 × 10^−^³	4.61	0.0689
BC	2.682 × 10^−^³	1	2.682 × 10^−^³	2.82	0.1372
A^2^	0.042	1	0.042	44.31	0.0003
B^2^	0.023	1	0.023	24.36	0.0017
C^2^	0.027	1	0.027	28.86	0.0010
Residual	6.666 × 10^−^³	7	9.522 × 10^−4^		
Lack of Fit	2.206 × 10^−^³	3	7.355 × 10^−4^	0.66	0.6185
Pure Error	4.459 × 10^−^³	4	1.115 × 10^−^³		
Cor Total	0.51	16			
Std. Dev.	0.031	R-Squared	0.9868		
Mean	0.79	Adj R-Squared	0.9699		
C. V. %	3.93	Pred R-Squared	0.9165		
PRESS	0.042	Adeq Precisior	21.968		

**Table 5:** ANOVA results of Box-Behnken experiments with zeta potential as the response parameter.

Source	Sum of Squares	df	Mean Square	F Value	*p*-Value (Prob > F)
Model	63.22	9	7.02	8.47	0.0051
A	5.27	1	5.27	6.35	0.0398
B	4.62	1	4.62	5.57	0.0503
C	30.46	1	30.64	36.73	0.0005
AB	0.59	1	0.59	0.71	0.4258
AC	1.38	1	1.38	1.66	0.2379
BC	1.00	1	1.00	1.21	0.3085
A^2^	2.78	1	2.78	3.35	0.1099
B^2^	7.85	1	7.85	9.46	0.0179
C^2^	10.08	1	10.08	12.15	0.0102
Residual	5.81	7	0.83		
Lack of Fit	4.54	3	1.51	4.79	0.0821
Pure Error	1.26	4	0.32		
Cor Total	69.03	16			
Std. Dev.	0.91	R-Squared	0.9159		
Mean	−9.61	Adj R-Squared	0.8078		
C. V. %	9.47	Pred R-Squared	−0.0813		
PRESS	74.64	Adeq Precisior	12.512		

**Table 6:** Response surface optimization results.

	OEMR1	OEMR2	OEMR3
PA-1	4.5	5.0	1.0
PA-2	2.2	1.2	5.0
PA-3	1.1	1.4	4.9
PA-4	2.3	3.7	5.0

**Table 7:** Results of quantitative Rietveld-Analysis of OEMR and PA-4.

Components of Crystals (%)	OEMR	PA-4
OEMR1	37.7	14.3
OEMR2	21.5	36.2
OEMR3	10.8	19.5
Rutile	30.0	30.0

## Data Availability

The raw data supporting the conclusions of this article will be made available by the authors on request.

## Supplementary Materials

The following supporting information can be downloaded at: https://www.mdpi.com/article/10.3390/ma17143605/s1. Table S1. Factors and levels of Box-Behnken experiments for EMR components ratio optimization for sludge dehydration. Table S2. Box-Behnken experimental design and CST results for EMR components ratio optimization for sludge dehydration. Table S3. Box-Behnken experimental design and SRF results for EMR components ratio optimization for sludge dehydration. Table S4. Box-Behnken experimental design and zeta potential results for EMR components ratio optimization for sludge dehydration.
