# Supplementary material for: Investigating the Dewatering Efficiency of Sewage Sludge with Optimized Ratios of Electrolytic Manganese Residue Components
Source: Materials (Basel). 2024 Jul 22;17(14):3605. doi: 10.3390/ma17143605 (PMC11278772; doi:10.3390/ma17143605)
Supplement: Supplementary file 1 [file materials-17-03605-s001.zip › materials-3088421-supplementary.docx]

**Table S1.** Factors and levels of Box-Behnken experiments for EMR components ratio optimization for sludge dehydration

| Factor | -1 | 0 | 1 |
| --- | --- | --- | --- |
| A | 1% | 3% | 5% |
| B | 1% | 3% | 5% |
| C | 1% | 3% | 5% |

The dosage of A, B, C is the percentage of the total sludge mass

**Table S2.** Box-Behnken experimental design and CST results for EMR components ratio optimization for sludge dehydration

| number | A | B | C | CST(s) |
| --- | --- | --- | --- | --- |
| 1 | 5 | 3 | 5 | 77.63 |
| 2 | 5 | 1 | 3 | 96.35 |
| 3 | 3 | 3 | 3 | 81.40 |
| 4 | 3 | 1 | 1 | 103.53 |
| 5 | 1 | 1 | 3 | 121.63 |
| 6 | 3 | 5 | 5 | 64.00 |
| 7 | 3 | 3 | 3 | 83.97 |
| 8 | 1 | 5 | 3 | 75.67 |
| 9 | 5 | 5 | 3 | 60.33 |
| 10 | 3 | 5 | 1 | 52.13 |
| 11 | 3 | 3 | 3 | 82.50 |
| 12 | 1 | 3 | 5 | 93.60 |
| 13 | 1 | 3 | 1 | 85.93 |
| 14 | 3 | 1 | 5 | 106.53 |
| 15 | 3 | 3 | 3 | 81.100 |
| 16 | 3 | 3 | 3 | 85.4 |
| 17 | 5 | 3 | 1 | 73.03 |

**Table S3.** Box-Behnken experimental design and SRF results for EMR components ratio optimization for sludge dehydration

| number | A | B | C | SRF (10^8^ s^2^·g^-1^) |
| --- | --- | --- | --- | --- |
| 1 | 5 | 3 | 5 | 0.6383 |
| 2 | 5 | 1 | 3 | 0.9812 |
| 3 | 3 | 3 | 3 | 0.7247 |
| 4 | 3 | 1 | 1 | 0.9416 |
| 5 | 1 | 1 | 3 | 0.8184 |
| 6 | 3 | 5 | 5 | 0.5802 |
| 7 | 3 | 3 | 3 | 0.7215 |
| 8 | 1 | 5 | 3 | 0.9504 |
| 9 | 5 | 5 | 3 | 0.9137 |
| 10 | 3 | 5 | 1 | 0.9625 |
| 11 | 3 | 3 | 3 | 0.7604 |
| 12 | 1 | 3 | 5 | 0.4567 |
| 13 | 1 | 3 | 1 | 0.9498 |
| 14 | 3 | 1 | 5 | 0.4558 |
| 15 | 3 | 3 | 3 | 0.7104 |
| 16 | 3 | 3 | 3 | 0.7910 |
| 17 | 5 | 3 | 1 | 0.9989 |

**Table S4.** Box-Behnken experimental design and zeta potential results for EMR components ratio optimization for sludge dehydration

| number | A | B | C | zeta potential (mV) |
| --- | --- | --- | --- | --- |
| 1 | 5 | 3 | 5 | -6.61 |
| 2 | 5 | 1 | 3 | -11.22 |
| 3 | 3 | 3 | 3 | -9.39 |
| 4 | 3 | 1 | 1 | -11.24 |
| 5 | 1 | 1 | 3 | -7.67 |
| 6 | 3 | 5 | 5 | -7.56 |
| 7 | 3 | 3 | 3 | -10.47 |
| 8 | 1 | 5 | 3 | -10.82 |
| 9 | 5 | 5 | 3 | -12.83 |
| 10 | 3 | 5 | 1 | -12.90 |
| 11 | 3 | 3 | 3 | -9.72 |
| 12 | 1 | 3 | 5 | -5.17 |
| 13 | 1 | 3 | 1 | -9.81 |
| 14 | 3 | 1 | 5 | -7.90 |
| 15 | 3 | 3 | 3 | -10.79 |
| 16 | 3 | 3 | 3 | -10.04 |
| 17 | 5 | 3 | 1 | -9.1 |
